# It’s all in the crystals…

**DOI:** 10.1107/S0907444911007797

**Published:** 2011-03-18

**Authors:** Zygmunt S. Derewenda

**Affiliations:** a Department of Molecular Physiology and Biological Physics, University of Virginia, Charlottesville, Virginia 22908-0793, USA

**Keywords:** macromolecular crystallography, crystals, crystallization, protein engineering, crystal contacts

## Abstract

Macromolecular crystallography relies on the availability and quality of single crystals; these are typically obtained through extensive screening, which has a very low intrinsic success rate. Crystallization is not a completely stochastic process and many proteins do not succumb to crystallization because of specific microscopic features of their molecular surfaces. It follows that rational surface engineering through site-directed mutagenesis should allow a systematic and significant improvement in crystallization success rates. Here, one such established strategy, surface-entropy reduction (SER), is discussed, including its successes, limitations and possible future developments.

## Abstract 2

Protein surface engineering is increasingly used as a routine tool to enhance the crystallization propensity of proteins. Future possibilities include the use of multi-site protein variants, rational modulation of solubility and the development of strategies to tackle membrane proteins.

## 1. Introduction

Single crystals constitute an essential prerequisite for structural investigations of biological macromolecules using X-ray diffraction. Good-quality high-resolution diffraction data virtually guarantee the success of structure determination and high precision of the resulting atomic model. The vast majority of problems encountered in crystal structure determination can typically be traced back to data-quality issues caused by crystal imperfections, poor resolution, unusual cases of pseudo­symmetry, anisotropic diffraction, twinning and so forth. Consequently, although the primary focus of structural biology is on the macromolecule that makes up a crystal, there is also considerable interest in the physical properties, nucleation and growth of the crystals themselves.

As vividly illustrated by the statistics assembled by various Structural Genomics Centers, in spite of considerable progress in the technology of liquid-handling and crystallization robotics, the overall success rate of canonical crystallization screening is low, ranging from at best 10–30% for small prokaryotic proteins to only a few percent for a representative range of eukaryotic proteins, including those from the human proteome (Page, 2008 ▶). However, these statistics do not reflect the true nature of the problem: many proteins do not succumb to crystallization in spite of extensive screening, while others may yield a variety of crystal forms in relatively few conditions. Crystallization is not really a completely stochastic process and is clearly determined by the microscopic nature of the protein’s surface. Thus, the modification of protein samples through recombinant methods constitutes an effective and rational approach to the problem of poor success rates in crystallization (Dale *et al.*, 2003 ▶).

It has long been recognized that variations in amino-acid sequences even among closely homologous proteins can cause dramatic differences in their ability to form crystals (Campbell *et al.*, 1972 ▶; Kendrew *et al.*, 1954 ▶; D’Arcy *et al.*, 1999 ▶). However, until recently it has not been possible to identify the exact relationship between specific microscopic surface features and the propensity of proteins to yield crystals. Instead, protein engineering was aimed primarily at protein stabilization and the removal of unstructured or unstable motifs that are likely to interfere with crystallization. I reviewed these methods recently (Derewenda, 2010 ▶). Although these strategies have helped with the crystallization of numerous important targets, they have not solved all of the problems. Numerous proteins that are made up of a single domain, fully folded and stable, are still recalcitrant to crystallization screens.

To address the crystallization bottleneck, several years ago we proposed a new strategy of surface engineering based on the concept of surface-entropy reduction (SER). Briefly, we argued that the transient protein–protein interactions that underlie nucleation and crystal growth are impeded by the loss of amino-acid side-chain entropy when the large and polar amino acids that are typically located on the protein’s surface are incorporated into crystal contacts with concomitant loss of degrees of conformational freedom. We hypothesized that mutating amino acids such as Lys, Glu and Gln to smaller amino acids such as Ala might create surface patches that are conducive to forming thermodynamically favourable inter­actions, ultimately forming crystal contacts (Longenecker *et al.*, 2001 ▶; Mateja *et al.*, 2002 ▶). The concept was validated experimentally using a model system, *i.e.* the globular domain of the human Lys- and Glu-rich protein RhoGDI (Rho-GTPase guanine nucleotide dissociation inhibitor), and then successfully used to obtain a plethora of novel crystal structures. These studies established that in general terms mutating two to three high-entropy residues situated next to or very close to each other in sequence yields the most successful outcome (Garrard *et al.*, 2001 ▶; Longenecker *et al.*, 2001 ▶). More recently, bioinformatics analyses showed that intermolecular contacts in known crystal structures are indeed depleted in high-entropy side chains (Cieślik & Derewenda, 2009 ▶) and that a high content of amino acids such as Lys and Glu correlates negatively with probability of crystallization (Price *et al.*, 2009 ▶). Surface-entropy reduction (or SER) has been the subject of several reviews (Derewenda, 2004 ▶, 2010 ▶; Derewenda & Vekilov, 2006 ▶). A server has been developed for the automated design of protein variants with enhanced crystallizability based on amino-acid sequence information (Goldschmidt *et al.*, 2007 ▶).

In this short article, I present an overview of what has been learned from the application of SER in numerous laboratories and comment on what future developments in this field might be expected.

## 2. The application and impact of SER

To date, more than 160 depositions of crystal structures based on crystals generated by the SER method have been made in the Protein Data Bank, attesting to the widespread popularity and success of this approach to crystallization. A gallery of most of the SER structures can be seen at the dedicated website http://ginsberg.med.virginia.edu/Ser/ and a review discussing these structures is in preparation. Among them are ∼60 novel proteins, seven protein–protein complexes, a significant number of protein complexes with small molecules used in drug design and two membrane proteins.

**Figure 1:**
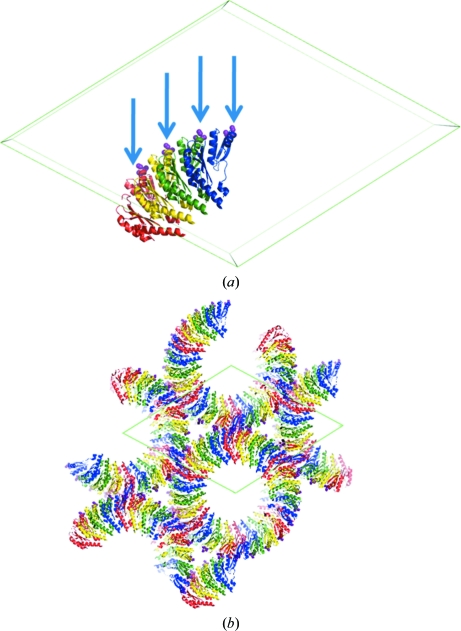
The crystal structure of the *Escherichia coli* EscJ protein, a molecular platform for type III secretion, solved using crystals with SER surface engineering (PDB entry 1yj7; Yip *et al.*, 2005 ▶). (*a*) The content of the asymmetric unit, an EscJ tetramer (each subunit is coloured differently), with the location of the three mutations in the solvent-exposed loop (E62A,K63A,E64A) indicated by arrows; the three alanines introduced by mutagenesis are shown as magenta spheres. (*b*) The assembly of the EscJ proteins into a 24-unit ring superstructure representative of the early stage of assembly of the type II secretion system (Yip *et al.*, 2005 ▶). Note that the mutated patches mediate the only crystal contacts, copied by the sixfold screw axis 6_5_, perpendicular to the plane of the diagram.

There are two obvious trends. First and foremost, the most significant impact of the SER strategy is on the crystallization of novel protein targets and their complexes that are recalcitrant to crystallization in the wild-type form. A number of biologically important high-profile structures have been solved using SER crystals. For example, the CUE–ubiquitin complex only succumbed to crystallization with a mutated CUE domain of Vps9p (Prag *et al.*, 2003 ▶). The structure offers critical insight into the conjugation of ubiquitin in numerous signalling pathways. SER also allowed for the crystallization of the intact Hsc70 chaperone and visualization of the interaction between its two domains, with important implications for the understanding of the allosteric mechanism (Jiang *et al.*, 2005 ▶). One of the most spectacular successes of SER was the crystallization of EscJ, a component of the type III secretion system, allowing crystal structure determination at 1.8 Å resolution (Yip *et al.*, 2005 ▶). This structure helps in understanding key aspects of virulence in Gram-negative pathogens (Fig. 1 ▶). A crystal structure of the ALIX/AIP programmed cell death 6-interacting protein is vital to the understanding of the mechanisms involved in retrovirus budding and endosomal protein sorting (Fisher *et al.*, 2007 ▶). The SER-engineered ALIX also made it possible to crystallize its complex with the YPX(n)L late domains of HIV-1 and EIAV (Zhai *et al.*, 2008 ▶). The crystal structure of the complex of c-Src with its regulator Csk obtained using a mutated variant of Csk provided a mechanistic explanation for the unusual specificity of Csk kinases (Levinson *et al.*, 2008 ▶). The complex of the NEMO Uban motif with diubiquitin, crystallized using an SER variant of NEMO (Rahighi *et al.*, 2009 ▶), provided an explanation for the detrimental effect of NEMO mutations in patients suffering from X-linked ectodermal dysplasia and immunodeficiency. Recently, an HIV-capsid component was crystallized by SER (Pornillos *et al.*, 2009 ▶); the structure will help in understanding the maturation of HIV and facilitate structure-based drug-design efforts.

The second application of SER, particularly in pharmaceutical companies, is to use the strategy to manipulate the target protein so as to generate crystal forms that are more suitable for drug discovery (*i.e.* higher resolution data, exposure of the active site to solvent) than those obtained for the wild-type protein. Among such successfully engineered drug targets are HIV reverse transcriptase (Bauman *et al.*, 2008 ▶; Das *et al.*, 2008 ▶) and beta-site amyloid precursor protein-cleaving enzyme (BACE-1), an important target in Alzheimer’s disease (Yang *et al.*, 2009 ▶; Rajapakse *et al.*, 2006 ▶; Coburn *et al.*, 2006 ▶). Many other investigations are under way but have not yet been published owing to intellectual property con­cerns.

## 3. What has SER taught us about crystal contacts and crystallization?

**Figure 2:**
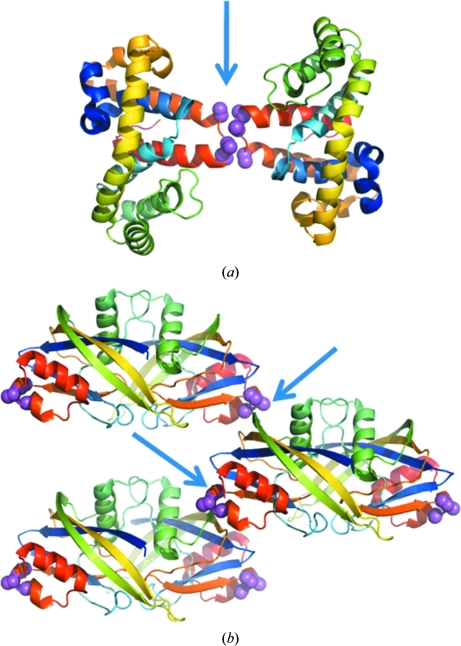
Two types of contacts commonly found in crystals obtained by SER engineering. (*a*) A homotypic symmetric contact creating a crystallo­graphic dimer in the RGSL domain of PDZ-RhoGEF (PDB entry 1htj; Longenecker *et al.*, 2001 ▶). Alanines replacing Lys463, Glu465 and Glu466 are shown as magenta spheres. The twofold axis is perpendicular to the plane of the drawing. (*b*) Heterotypic contacts (arrows) that mediate interactions between noncrystallographic dimers of a putative NTP pyrophosphorylase (PDB entry 3n77). The dimer interface is mediated by a wild-type surface.

The vast majority (>90%) of crystals obtained through SER show that the crystal contacts are intimately mediated by the mutated surface patches. There are two principal variations in this respect: the contacts can be homotypic (*i.e.* two identical patches interact across an interface generated by a crystallo­graphic or noncrystallographic dyad) or heterotypic (*i.e.* where the mutated patch interacts with a different surface patch on an adjacent molecule; this is typically observed for molecules related by translation or screw axes; Fig. 2 ▶). It is obvious from these data that the SER strategy does in fact allow direct engineering of crystal contacts by creating surface patches that are significantly more conducive to cohesive interactions than the wild-type molecular surface. Several distinct mechanisms may be in play. Firstly, as suggested by the original hypothesis, surface patches depleted in high conformational entropy residues might preferentially form thermodynamically favourable crystal contacts. Also, in the absence of large flexible side chains the solvent-accessible backbone amide and carbonyl groups have a higher potential to form hydrogen bonds to water molecules and organise a network of ordered solvent which is released upon nucleation, with additional entropy gain. Release of water from the protein’s surface is the primary entropic driving force for crystallization (Vekilov, 2003 ▶; Vekilov *et al.*, 2002 ▶). Moreover, the exposed backbone may mediate direct intermolecular hydrogen bonds, conferring stereochemical specificity on crystal contacts. Finally, patches created by alanines or other small aliphatic amino acids generate cohesive interactions through the hydrophobic effect.

All these phenomena are quite close in their nature to those that govern biologically relevant intermolecular interfaces. It has been argued in the past that crystal contacts and biological interfaces are sufficiently distinct in structure to allow largely automated discrimination and annotation (Bahadur & Zacharias, 2008 ▶; Bahadur *et al.*, 2004 ▶; Ofran & Rost, 2003 ▶). Biological interfaces tend to be significantly larger than average crystal contacts and their amino-acid composition shows more pronounced deviations from random patches than that in crystal contacts. All of this is true, but these analyses overlook the fact that not all of the crystal contacts, as identified simply by physical proximity, must in fact be *cohesive* and thermodynamically *relevant* contacts. Molecules can be brought within physical contact in the incipient crystal nucleus in serendipitous ways, driven by the thermodynamic ‘collapse’ of the nucleus. Our preliminary investigation shows that crystal contacts can either be cohesive and contribute to the thermodynamic stability and growth of the crystals (*i.e.* primary contacts) or they can be repulsive and forced (secondary contacts). Because all analyses to date assume that physical proximity (*e.g.* distance criterion) determines all crystal contacts, the outcome of these studies may be biased and therefore may not faithfully reveal the exact nature of cohesive primary crystal contacts.

## 4. The current and potential success rate of SER

An often-asked question is how much does SER increase the probability of obtaining crystals? Unfortunately, there is no unambiguous answer. In the only truly systematic study of the application of SER to diverse human targets, it has been found that SER rescued three out of the 20 tested proteins (15%) that did not crystallize in their wild-type form (A. Edwards, University of Toronto, personal communication). It should be recognized, however, that virtually all of the applications of SER involve the engineering of a single surface patch, with a maximum of three amino acids mutated to Ala. In approximately half of these structures the mutated patches form homotypic contacts (see above, §3), leading to crystallographic homodimers related by a twofold axis or a noncrystallographic dyad. It is possible that even transient dimerization significantly increases the propensity of the protein to crystallize (Banatao *et al.*, 2006 ▶). However, this means that once such a homodimer forms the engineered patch has no further role to play and other wild-type surface patches must provide suitable intermolecular contacts for the formation of a three-dimensional crystal. If no such suitable patches are present then crystallization will not occur. This strongly suggests that engineering of second- and third-order cohesive contacts may be necessary to bring the success rate of SER closer to the highly desired 100% range. An interesting recent study indeed supports this notion: the human vaccinia-related kinase 1 (PDB entry 3op5) was crystallized after *four* patches, con­taining a total of 11 mutations, had been introduced based on the *SERp* server predictions. The asymmetric unit of these crystals contains four molecules; while one set of mutations is located in the disordered C-terminus, the other three each mediate unique crystal contacts. This example clearly shows that SER can and should be developed further so that in principle any macromolecule can be coerced to form crystals mediated by a set of engineered contacts.

## 5. Can SER be helpful for membrane-protein crystallization?

It is particularly encouraging that SER is being successfully applied to membrane proteins, although there are only two such examples to date: the complex of the K^+^-gated channel KChIP1 with the Kv channel-interacting protein (Kv4.3 T1) crystallized using a double mutant K(160,167)A of the latter (Pioletti *et al.*, 2006 ▶) and the impressive crystal structure of the BetP Na^+^/betaine symporter obtained using crystals of a triple variant E(44,45,46)A (Ressl *et al.*, 2009 ▶). In the latter case the mutations occur within a disordered fragment of the structure and do not participate in the crystal contacts.

In the general case SER is not directly applicable to membrane proteins. The SER strategy assumes that the protein’s surface is uniformly populated with large polar amino acids and this assumption breaks down for membrane proteins. Ironically, the transmembrane portions of membrane proteins typically have an amino-acid composition that is much closer to that desired for crystal contacts, but in this case these nonpolar surfaces are responsible for aggregation and are shielded by detergents. The remaining solvent-exposed surfaces are small and offer very limited opportunities for engineering. Moreover, while the loss of a few polar amino acids in a globular protein does not compromise its solubility (see below), in a membrane protein such mutations are not expected to have only benign consequences. Thus, other approaches might be more suitable (see §[Sec sec7]7).

## 6. SER and protein solubility

**Figure 3:**
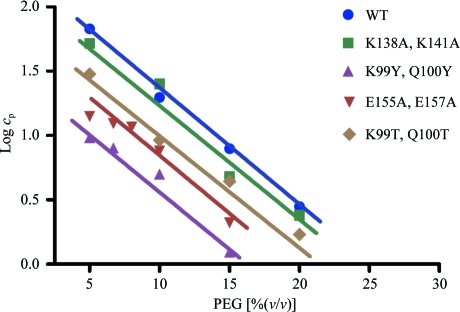
Solubility data in PEG 6000 for selected mutants of RhoGDI (Cooper *et al.*, 2007 ▶) plotted on a logarithmic scale following Cohn’s equation, log*c*_p_ = *B* − *Kc*, where *c*_p_ is the protein concentration, *c* is the precipitant concentration, *B* is the idealized protein solubility at *c* = 0 and *K* is a protein-dependent constant. For all mutants, the solubility in PEG 6000 is systematically lowered and follows the pattern observed by Trevino *et al.* (2007 ▶). Importantly, the *K* parameter appears unchanged, so that the solubility lines are approximately parallel. The replacement of lysines by alanines causes only a marginal decrease in solubility; the effect is more significant when threonines are introduced and is the largest with tyrosines. Replacing glutamates by alanines causes a significantly larger solubility reduction. Solubility was measured at room temperature at pH 8.0 (buffered) as a function of PEG 6000 concentration.

When the SER concept was first published, it was suggested that mutations of the K→A and E→A type are bound to decrease the protein solubility and thereby promote crystallization indirectly. However, the fact that mutated patches are almost invariably involved in mediating crystal contacts (see above) attests to the contrary. Solubility is a macroscopic property and while it is determined by the amino-acid content (or more precisely the surface amino-acid composition), it is not directly correlated with local microscopic surface structure. To better understand the impact of specific surface mutations on solubility, we initiated systematic investigations of the solubility of select SER mutants of RhoGDI (these are currently being prepared for publication). Fig. 3 ▶ shows representative data for the solubility of four double mutants in polyethylene glycol. It is clear that the replacement of two neighbouring lysines by alanines causes only a marginal reduction in solubility; the replacement of glutamates by alanines is more significant but not dramatic. In contrast, the replacement of a lysine and a glutamine by two tyrosines reduces the solubility of the protein by an order of magnitude. Our results are consistent with the recent systematic study of the impact of surface mutations on protein solubility using ribonuclease as a model system that shows that the replacement of Lys by Ala or Ser does not reduce solubility; in fact, Ser mutants should be more soluble than the wild-type protein (Trevino *et al.*, 2007 ▶). Further studies will allow the design of mutations that simultaneously enhance crystallizability and modify solubility in a desired way.

## 7. SER-enhanced chaperones

One of the exciting new applications of SER is the engineering of proteins that serve as crystallization chaperones. Two complementary strategies are possible. Firstly, it has been established for some time that fusion proteins containing a carrier protein and the target molecule can sometimes crystallize more effectively owing to the carrier protein, which may mediate some or most of the crystal contacts. Various such carrier proteins have been tried, but maltose-binding protein (MBP) has been the most successfully used molecule (Smyth *et al.*, 2003 ▶). Recently, it has been suggested that an engineered MBP might be even more effective in promoting the crystallization of a fusion protein, and indeed a D82A, K83A, K239 variant was successfully utilized to crystallize the RACK1 protein (Ullah *et al.*, 2008 ▶). The other option is to use phage display to generate recombinant Fab fragments or other engineered scaffolds (Koide, 2009 ▶; Gebauer & Skerra, 2009 ▶). These scaffolds could also be engineered for enhanced crystallizability by SER, although this has not yet been achieved.

## 8. Concluding remarks

The SER strategy has convincingly demonstrated that macromolecular crystal engineering is a useful and effective tool with the promise of improving overall crystallization success rates well above those stemming from pure canonical screening. However, it is also clear that more elaborate approaches will be required in order to tackle the most complex problems such as the crystallization of membrane proteins or large complexes. Work on these questions is in progress.
